# America's Awakening to Hospital Values: The Spread of Efficiency

**Published:** 1917-04-28

**Authors:** 


### AMERICA'S AWAKENING TO HOSPITAL VALUES

### The Spread of Efficiency.

One of the most remarkable evidences of the spread of efficiency and awakened interest in hospitals, and of developments which aim at extending and improving facilities of all kinds for the training, treatment, uplifting, and restoration of dependants of all types, is afforded by the magnificent growth and extended influence for good of the American Hospital Association. In the last twelve years this Association has grown from a membership of a few hundreds to one of thousands, and the attendance at its Annual Conference brings together representatives of all types of institutions, and, through the careful selection of subjects and of the readers of papers to introduce them, it promises soon to become of increasing practical value, directly to the whole of the workers in the American hospital field, and, indirectly, to the whole population of the United States. America prides herself upon being democratic to the core, and it is to the democratic basis on which the American Hospital Association has rested since the Boston meeting in 1906, that the Association owes its ever-extending membership and widening interest and influence.

The establishment of *The Modern Hospital*, which has rapidly developed into a monthly magazine, demy quarto in size, containing some 200 pages, of which some 120 contain advertisements and eighty are devoted to literary matter, illustrations, and plans, is a wonderful instance of rapidity of growth in magazine literature. The editors of *The Modern Hospital* are Dr. Henry M. Hurd, of the Johns Hopkins Hospital, Baltimore; Dr. Washburn, of the Massachusetts General Hospital, Boston; Dr. Winford H. Smith, Johns Hopkins Hospital; Dr. S. S. Goldwater, Mount Sinai Hospital, New York; Dr. W. L. Babcock, Grace Hospital, Detroit; and Dr. John A. Hornsby, who occupies the editorial offices in Conway Building, Chicago. The editors thus include the best-known and most deservedly trusted of hospital administrators in the United States. Dr. Hornsby is a very influential man, of many parts and excellent qualities. He has a way with him which makes him a model chairman of a large meeting, for he possesses the knack of keeping everybody in the best of humour, and of getting the business through with despatch to the satisfaction of everyone. *The Modern Hospital* is to be congratulated upon his occupancy of the editorial chair at its central office.

The plan of the paper is to devote the space before the leading articles mainly to the history, organisation, practical work, plans, with information of many kinds relating to hospitals and institutions for the relief and treatment of disease in all its aspects. Some four pages are devoted to editorials. Ample space is given to every branch of hospital work, including the remodelling of a hospital, electricity as applied to hospitals, tuberculosis in every form and under varied conditions, current hospital literature (a strong and excellent department), and a Department of Nursing conducted by Miss Annie W. Goodrich, a most devoted and able worker, and knowledgeable too. Then there are an immensity of special subjects, including Maternity, the Modern Sanatorium, Social Hygiene, Foreign Correspondence, a Department of Dietetics, Industrial Welfare, Queries and Answers, the Bulletin of the American Hospital Association, Book Reviews, New Instruments and Equipments, and news of the hospital field in short paragraphs spread over many of the advertisement pages.

We hope that the removal of the editorial department to new and more suitable offices, and the increase in the staff, will, with Dr. Hornsby's usual energetic and practical assistance, speedily result in all plans published in *The Modern Hospital* being reduced to scale, on the plan pursued by The Hospital for over thirty years. It would be a very pleasant and helpful thing if our brother editor were to join hands with us by making this change, for that would make the files of *The Modern Hospital* with those of The Hospital indispensable to every architect associated with hospital construction. The educational effect and value which would follow cannot be exaggerated. We may take this opportunity to express our personal indebtedness for the great courtesy and help extended to us by Dr. Hornsby and the President of *The Modern Hospital* during our visit to the United States last fall. Nothing could exceed their courtesy, and gratitude makes us hope that they may give us an opportunity one day to welcome them to the Old Country, and to do what we can to add to the enjoyment and practical value of their enterprise by every means in our power.

